# Effects of a Tailored Pediatric Rehabilitation Protocol on Children With Neurometabolic Disorder: A Case Report

**DOI:** 10.7759/cureus.57115

**Published:** 2024-03-28

**Authors:** Neha A Brahmane, H V Sharath, Nikita H Seth, Arasha F Khan

**Affiliations:** 1 Department of Paediatric Physiotherapy, Ravi Nair Physiotherapy College, Datta Meghe Institute of Higher Education & Research, Wardha, IND; 2 Department of Neurophysiotherapy, Ravi Nair Physiotherapy College, Datta Meghe Institute of Higher Education & Research, Wardha, IND

**Keywords:** metabolic disorder, pediatric physiotherapy, rehabilitation, physical therapy, neurometabolic disorder

## Abstract

Neurometabolic disorders encompass a diverse group of conditions characterized by inborn errors of metabolism, affecting various aspects of neurological function. This case report focuses on an 11-year-old male child with a neurometabolic disorder who presents with walking difficulties, speech impairment, and neurological symptoms. The background emphasizes the heterogeneity of neurometabolic disorders, their genetic and clinical complexity, and the need for tailored interventions to address specific manifestations. The primary aim of this study is to implement a comprehensive physiotherapy intervention plan for an 11-year-old male child with a neurometabolic disorder, targeting the improvement of gait abnormalities, regaining developmental milestones, and addressing associated challenges such as fatigue. The physiotherapy intervention plan employs a multifaceted approach, incorporating principles of neuroplasticity, motor learning, and adaptive strategies. A comprehensive gait training protocol involves proper orthotic fitting, forearm support walker training, treadmill exercises, and parallel bar training. Developmental milestones are addressed through motor and fine motor skill exercises. Coping strategies and energy conservation techniques are integrated to manage fatigue. The study utilizes outcome measures, including the Gross Motor Function Measure, Manual Ability Classification System, and Functional Independence Measure, to assess the impact of the intervention on the patient’s functional abilities. Preliminary findings suggest promising improvements in gait, motor skills, and overall functional independence following the implemented physiotherapy intervention. The study underscores the potential effectiveness of a tailored approach rooted in neuroplasticity and motor learning principles for individuals with neurometabolic disorders. The patient-centered care model, encompassing coping strategies and energy conservation techniques, contributes to holistic well-being. While specific literature on these interventions in neurometabolic disorders is limited, the study provides valuable insights and calls for further research to refine and expand tailored therapeutic approaches in this challenging clinical context.

## Introduction

Neurometabolic diseases are a group of diseases that are categorized as the dysfunction of the lack of vitamins that are necessary for the human body to carry out a specific chemical reaction. The incidence of neurometabolic disorders is 1 in 1,000 live births. Such disorders, at different ages, could manifest as sepsis, hypoglycemia, and other neurologic disorders [1]. These are the progressively inborn errors of metabolism, and many are treatable. These groups are heterogeneous genetically as well as clinically [2]. Neurometabolic disorders are a group of different characteristic features that include movement disorders, epilepsy, ataxia, hypotonia, pyramidal signs, and psychomotor retardation with or without regression [3]. There is a high range of metabolic disturbances seen, which is a result of clinical syndrome, with a prevalence of 1 in 1,000 live births [4].

As per the classification, neurometabolic disorders are classified into three categories: intermediary disorders, complex disorders, and neurotransmitter disorders. Certain parts of the central or peripheral nervous systems may be specifically affected by these disorders. This denotes an array of indications and manifestations associated with the impacted areas, specifically, the basal ganglia, spinal cord, extrapyramidal tract, and lower motor unit [5].

In neurometabolic disorder, it has also been identified that there is a possible involvement of mitochondrial disorder and dysfunction, which also includes the theory of a high level of exposure to oxidative stimuli due to which various toxins cause metabolic abnormalities leading to impairment of normal cellular function [6]. Metabolic disorders that affect a child’s brain are considered complex conditions. The mechanism that leads to damage to the structure is complicated, and identification and diagnostic imaging are nonspecific [7].

However, delayed or false diagnosis further leads to irreversible injury to the brain. There is a special consideration applied to the genotype in neurometabolic disorders like phenocopies that exhibit similar characteristics that are caused by mutations of other and unrelated genes [8]. As mentioned before, the discovery of the gene is accelerating, and the pathophysiological mechanism of the abovementioned disorder is still idiopathic. A few stated, not necessarily mutually exclusive, theories state the buildup of metabolic products and the negative feedback regulation of enzymes [9]. There is a crucial role played by physiotherapy in the management of neurometabolic disorders. That aims to treat neurological as well as musculoskeletal complications [10]. Although the neurometabolic reasons for dystonia are varied and often difficult to identify, many of these conditions can be treated [11].

In addition to ataxia, mental deterioration, unusual conduct, and sleep difficulties, children may exhibit an irregular walk. Abnormal tones, spasticity or hypotonicity, and rapid deep tendon reflexes can all be seen during a neurological examination. Children may also exhibit cerebellar symptoms, poor attention, verbal disturbance, and visual loss. Aggression, mood problems, and behavioral abnormalities are the predominant signs of neurometabolic disorders in adults. These conditions are primarily psychiatric in nature [12,13]. In early infancy and childhood, the growth of the body and the maturity of the bodily systems that are involved in movement set boundaries for and specify functional skills [14]. Reduced gait speeds may have an adverse effect on their quality of life and ability to participate in the community [15,16]. The organization of information from the three sensory systems - visual, vestibular, and somatosensory - as well as the selection of the appropriate sensory signals to produce coordinated movements are necessary for maintaining body balance [17,18]. The objective of the current study is to investigate the impact of the tailored pediatric rehabilitation protocol on the quality of life of children with neurometabolic disorders, as reported by both the children and their caregivers.

## Case presentation

Patient information

This is the case of an 11-year-old male child who appeared healthy as he achieved developmental milestones accordingly. Six years ago, the child began experiencing difficulty walking, characterized by swaying while walking and frequent falls, along with a scissoring walking pattern. Three to four years ago, he also exhibited speech difficulties and a history of saliva drooling, coinciding with the year of his parents’ consanguineous marriage. Subsequently, the child was taken to a local hospital in Shirdi, where an MRI investigation revealed a lesion in the bilateral parieto-occipital region, accompanied by regressive milestones.

Ayurvedic medications were administered, but with no discernible improvement, the treatment was discontinued after six months. The child was then referred to the Acharya Vinoba Bhave Rural Hospital for further management, presenting with complaints of difficulty walking, swallowing, and speaking, along with saliva drooling and white-colored lesions on the face. Further investigations, including an MRI brain plain and contrast on November 25, 2023, suggested an acquired demyelinating disorder. Lactate and CSF analyses yielded normal findings, while ceruloplasmin levels were within the normal range and alkaline phosphate levels were reduced. The pathology report indicated an elevated level of lymphocytes.

Ultimately, the child was diagnosed with a neurometabolic disorder, along with atopic dermatitis and acute malnutrition.

Clinical findings

The patient’s informed consent was obtained before the examination, following which a physical assessment was conducted. Throughout the assessment, the patient’s vitals remained stable, with the highest attainable functional position being sitting without support. Physically, the patient presented as mesomorphic, exhibiting slurring of speech. A detailed timeline of events is provided in Table 1.

**Table 1:** Timeline of events AVBRH, Acharya Vinoba Bhave Rural Hospital

Events	Timeline
Difficulty walking	2017
Difficulty in speech	2019
Drooling of saliva	2022
Difficulty swallowing	2022
Ayurvedic medication	2023
AVBRH	2023

Active movements of both the bilateral upper and lower limbs were observed, with all developmental milestones achieved as expected. However, a regressive milestone was noted. Muscle tone was assessed using the modified Ashworth scale, as detailed in Table 2.

**Table 2:** Assessment of muscle tone using the modified Ashworth scale Grade 0: no increase in tone; Grade 1: slight increase in tone, manifesting as a catch when the limb is moved in flexion or extension; Grade 1+: slight increase in tone, with a catch followed by minimal resistance throughout the ROM; Grade 2: marked increase in tone, with most limbs easily flexed; Grade 3: considerable increase in tone, making passive movement difficult; Grade 4: limb rigid in flexion or extension ROM, range of motion

Muscles	Right	Left
Shoulder muscles	1+	1+
Elbow muscles	1+	1+
Wrist muscles	1+	1+
Hip muscles	2+	2+
Knee muscles	2+	2+
Ankle muscles	2+	2+

The range of motion of the upper and lower limbs was normal. Sensory examination revealed intact sensation. Bilateral tendoachilles tightness was observed. Constipation was noted in bowel function, while bladder function appeared normal. Positive cerebellar signs, including past pointing, dysdiadochokinesia, and pendular knee jerk, are detailed in Table 3.

**Table 3:** Positive cerebellar signs in the patient ++: normal; +++: hyperactive without clonus

Reflexes	Right	Left
Bicep jerk	++	++
Tricep jerk	++	++
Supinator jerk	++	++
Knee jerk	+++	+++
Ankle jerk	+++	+++
Plantar response	Extensor response	Extensor response

Diagnostic investigations

**Figure 1:**
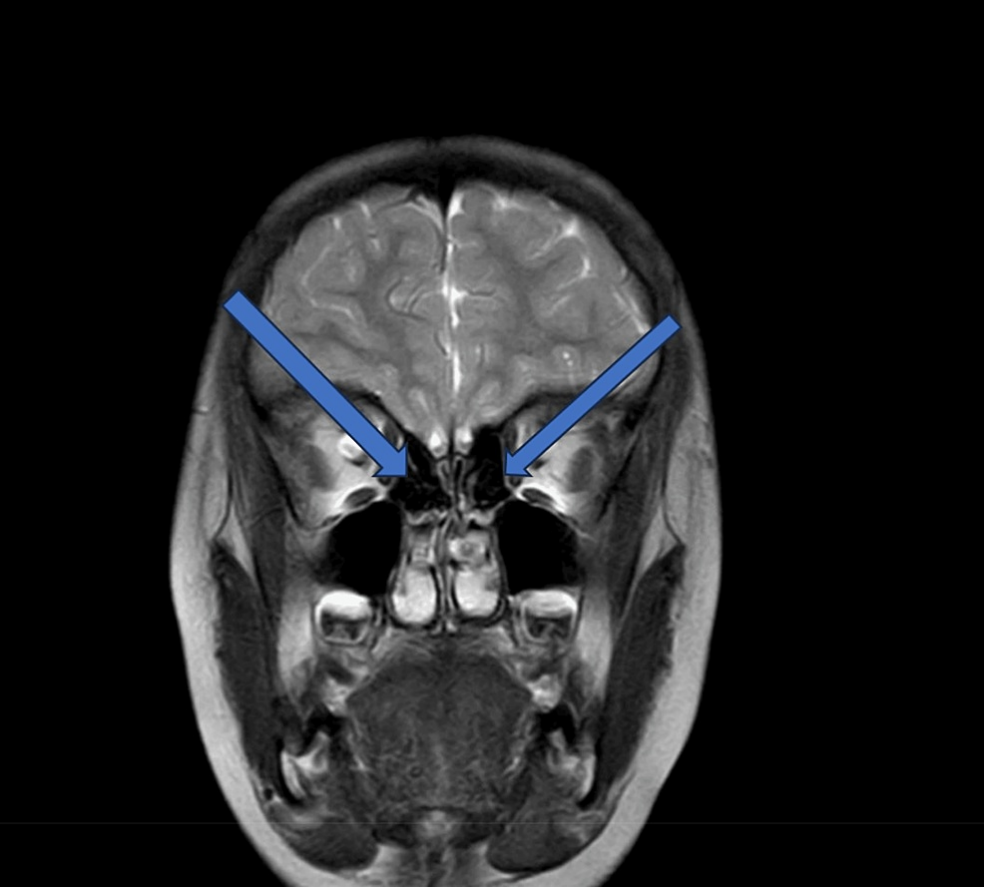
MRI image depicting findings suggestive of an acquired demyelinating disorder in the brain

MRI brain plain and contrast revealed evidence of T2 and flair subtle hyperintensity involving bilateral centrum semi-ovale. Prominent extra-axial CSF space was noted as suggestive of diffuse cerebral and cerebellar atrophy. These features suggested an acquired demyelinating disorder (Figure 1).

Table 4 presents details of the physiotherapeutic intervention for the patient.

**Table 4:** Physiotherapy intervention for the patient AFO, ankle foot orthosis

Serial number	Goal	Exercise
1	To reduce spasticity	Roods inhibitory techniques: sustained stretch, joint approximation, and ring sitting
2	To improve muscle strength	Upper extremity: small ball-catching and throwing activities, resistive isometric exercises, and pegboard activities. Lower extremity: pelvic bridging exercises and abdominal curl exercises. Quadripod: weight bearing with perturbations, ball-kicking activity, and sitting position
3	To regain developmental milestones	Motor skills: quadripod – weight bearing. Quadripod – perturbations anterior-posterior and lateral shifts; lateral shifts in sitting (left to right and right to left); sit-to-stand activity: initially weight bearing on the affected leg. Fine motor skills: squeezing gel ball, pegboard activities, block building, and coloring and painting activities
4	To improve balance and coordination	Task-oriented approach: reach-outs in quadripod positions with perturbations, reach-outs in multiple directions while sitting, picking up objects from the floor, and ball kicking
5	To promote gait training	Pre-gait training parameters: Proper orthotic fitting including Static AFO and prescription of walker. Gait training utilizing a forearm support walker with Static AFO in overground training. Gait training on a treadmill utilizing a body support harness. Gait training using parallel bars and mirror biofeedback
6	To teach coping strategies	Energy conservation techniques: proper placement of essential items near the bed and bathroom, with the room ideally located on the same floor to minimize movement. Minimization of stair usage and utilization of proper stair climbing and descending techniques, synchronized with controlled breathing. Adequate sleep of six to eight hours at night and one- to two-hour rest periods during the day to replenish energy levels. Pacing techniques: integration of breathing exercises every 20-25 minutes between physiotherapy training or exercise sessions to uphold optimal pacing and energy management

Outcome measures

Various measures are employed to assess physical function, gait, coordination, and daily activity. These assessments were initially conducted on the first day of treatment and again at the conclusion of the hospital stay, spanning 20 days.

The Gross Motor Function Measure (GMFM) is a standardized observational instrument used to evaluate and record changes in gross motor function over time in children with cerebral palsy or other neurological impairments. It assesses activities such as lying and rolling, sitting, crawling, standing, walking, running, and jumping. The GMFM provides valuable information for treatment planning, goal setting, and tracking progress in rehabilitation programs.

The Manual Ability Classification System (MACS) is a classification system used to describe how children with cerebral palsy use their hands to handle objects in daily activities. It categorizes manual abilities into five levels based on the child’s typical performance, ranging from Level I (able to handle objects easily and successfully) to Level V (limited ability to handle objects). The MACS helps healthcare providers and therapists better understand a child’s manual abilities, plan interventions, and communicate effectively about the child’s functional abilities with parents and other professionals.

The Functional Independence Measure (FIM) is a widely used assessment tool for measuring functional status and independence in individuals with disabilities, particularly those undergoing rehabilitation. It evaluates the individual’s ability to perform activities of daily living and mobility tasks, such as eating, grooming, bathing, toileting, dressing, walking, and transferring. The FIM consists of 18 items, each scored on a scale from 1 (total assistance) to 7 (complete independence), providing a comprehensive assessment of the individual’s functional capabilities and guiding rehabilitation planning and discharge decisions (Table 5).

**Table 5:** Outcome measures pre- and post-treatment The data has been represented as N. GMFM score 0-25: severe gross motor impairment; 26-50: moderate gross motor impairment; 51-75: mild gross motor impairment; 76-100: minimal to no gross motor impairment MACS score Level I: handles objects easily and successfully; Level II: handles most objects but with some difficulty; Level III: handles objects with difficulty and needs help to prepare and/or modify activities; Level IV: handles a limited selection of easily managed objects in adapted situations; Level V: does not handle objects and has severely limited ability to perform even simple actions FIM score 18-36: severe functional dependence; 37-54: moderate functional dependence; 55-72: mild to moderate functional dependence; 73-90: mild functional dependence; 91-108: near independence; 109-126: complete independence FIM, Functional Independence Measure; GMFM, Gross Motor Function Measure; MACS, Manual Ability Classification System

Outcome measures	Pre-treatment	Post-treatment
GMFM	83.4%/100%	70%/100%
MACS	Level II/Level V	Level I/Level V
FIM	104/126	110/126

## Discussion

The presented case highlights a complex scenario involving an 11-year-old male child with neurometabolic disorder presenting with various neurological symptoms such as difficulty walking, speech impairment, and lesions over the face. The patient’s history includes consanguineous marriages between parents, which might be indicative of a genetic component to the disorder. Diagnostic investigations, including MRI brain plain and contrast, revealed evidence suggestive of an acquired demyelinating disorder with diffuse cerebral and cerebellar atrophy. Additionally, laboratory findings showed normal lactate and CSF levels but abnormalities in ceruloplasmin, alkaline phosphatase, creatinine, and increased lymphocytes, indicating metabolic disturbances.

The neurometabolic disorder is majorly associated with gross motor disabilities in children, which leads to an inability to perform daily living activities. Mat exercises have a huge impact on effective treatment for improving balance, strength, and postural control, as they purely work to strengthen core muscles. Gross motor and physical activity, which included mat exercises, proved a promising alternative to intense training, and it also created interest among children.

In previous studies, it has been proven that strength has an improvement in maximizing work resistance and gait, improving gross motor functions, and improving standing balance in pediatrics as well as adolescents, but simultaneously it has shown a short-duration effect, which urges the importance of high-intensity training [19]. The task-oriented approach places a strong emphasis on the creation of “functional tasks” and values the connection between people, tasks, and the environment in which they are completed. Children with cerebral palsy are able to actively attempt to solve issues in functional tasks, adjust to changes in their surroundings, and apply the functions they have learned in training to real-world situations. Through the continuous formation of new neural connections and neural networks, the brain is able to recreate cortical motor maps [20].

The incorporation of Roods inhibitory techniques for spasticity reduction, such as sustained stretch, joint approximation, and ring sitting, aligns with established physiotherapeutic practices aimed at improving muscle tone and flexibility. Sustained stretching involves maintaining a prolonged stretch on a muscle or group of muscles. This technique aims to stimulate Golgi tendon organs, which are sensory receptors located in the musculotendinous junction. Activation of Golgi tendon organs induces an inhibitory reflex that decreases muscle tension and spasticity [21]. Joint approximation involves providing compression or traction at a joint. This technique stimulates joint receptors, including Pacinian and Ruffini corpuscles. The activation of these receptors results in inhibitory effects on muscle tone, contributing to the reduction of spasticity [22]. Ring sitting induces rotational movements through the spine, providing varied sensory input to the muscles and joints. This technique influences muscle spindle activity and facilitates the reciprocal inhibition of antagonist muscles, contributing to the reduction of spasticity [23].

The mechanisms for regaining developmental milestones described above collectively leverage the principles of neuroplasticity and motor learning to facilitate the regaining of developmental milestones. These interventions aim to create a neurologically enriched environment, foster adaptive changes in the nervous system, and promote the acquisition of functional skills. While specific studies on these mechanisms in the context of neurometabolic disorders might be limited, the referenced principles are widely recognized in the broader fields of neuroscience and rehabilitation [24,25].

Initiating gait training with a forearm support walker offers stability and support, facilitating weight shifting and coordinated movements. This stepwise progression helps build confidence, improve balance, and foster the development of a more functional gait pattern [26]. Treadmill exercises provide a controlled environment for gait training, allowing for focused work on specific gait parameters. The use of a body support harness enhances safety and reduces the fear of falling, while the repetitive nature of treadmill exercises promotes motor learning and adaptability of the gait pattern [27]. Coping strategies involve the development of adaptive techniques to manage challenges associated with neurometabolic disorders. This may include addressing psychological aspects, such as stress and anxiety, as well as developing cognitive-behavioral approaches to overcome obstacles in daily life. Coping strategies empower individuals to effectively deal with the emotional and physical aspects of their condition [28].

Limitations

Limitations of the study include the potentially insufficient timeframe to capture the long-term effects of the physiotherapy intervention, suggesting that prolonged follow-up periods would offer a more comprehensive understanding of sustained benefits. Additionally, the absence of extended follow-up data impedes insights into the durability of observed improvements, highlighting the need for longer-term assessments to clarify the intervention’s lasting impact. Moreover, the study’s limited sample size, focusing solely on a single 11-year-old male child, restricts the generalizability of the findings to a broader population.

Implications

The individualized and creative approach demonstrated in the physiotherapy intervention plan underscores the importance of tailoring rehabilitation strategies to the unique needs of pediatric patients with neurometabolic disorders. However, limitations identified in the study, such as a small sample size and the absence of a control group, point to areas for future research. Training programs for physiotherapists and healthcare professionals should integrate principles of neuroplasticity, motor learning, and adaptive strategies. This would equip practitioners with the skills needed to design and implement effective, individualized interventions for pediatric patients with neurometabolic disorders, thereby advancing the field and improving patient outcomes.

## Conclusions

The tailored approach, rooted in neuroplasticity and motor learning principles, demonstrates potential effectiveness in enhancing overall motor as well as functional independence. The comprehensive gait training, motor skill exercises, and integration of coping strategies and energy conservation techniques contribute to a holistic approach that showed improvement. Measured outcomes indicate positive results, highlighting the significance of individualized care for individuals with neurometabolic disorders.
